# Delta opioid receptor on equine sperm cells: subcellular localization and involvement in sperm motility analyzed by computer assisted sperm analyzer (CASA)

**DOI:** 10.1186/1477-7827-8-78

**Published:** 2010-06-25

**Authors:** Maria Albrizio, Giovanni M Lacalandra, Elisabetta Micera, Antonio C Guaricci, Michele Nicassio, Antonia Zarrilli

**Affiliations:** 1 Department of Animal Production, Faculty of Veterinary Medicine, University of Bari, I-70010, Valenzano (BA), Italy

## Abstract

**Background:**

Opioid receptors and endogenous opioid peptides act not only in the control of nociceptive pathways, indeed several reports demonstrate the effects of opiates on sperm cell motility and morphology suggesting the importance of these receptors in the modulation of reproduction in mammals. In this study we investigated the expression of delta opioid receptors on equine spermatozoa by western blot/indirect immunofluorescence and its relationship with sperm cell physiology.

**Methods:**

We analyzed viability, motility, capacitation, acrosome reaction and mitochondrial activity in the presence of naltrindole and DPDPE by means of a computer assisted sperm analyzer and a fluorescent confocal microscope. The evaluation of viability, capacitation and acrosome reaction was carried out by the double CTC/Hoechst staining, whereas mitochondrial activity was assessed by means of MitoTracker Orange dye.

**Results:**

We showed that in equine sperm cells, delta opioid receptor is expressed as a doublet of 65 and 50 kDa molecular mass and is localized in the mid piece of tail; we also demonstrated that naltrindole, a delta opioid receptor antagonist, could be utilized in modulating several physiological parameters of the equine spermatozoon in a dose-dependent way. We also found that low concentrations of the antagonist increase sperm motility whereas high concentrations show the opposite effect. Moreover low concentrations hamper capacitation, acrosome reaction and viability even if the percentage of cells with active mitochondria seems to be increased; the opposite effect is exerted at high concentrations. We have also observed that the delta opioid receptor agonist DPDPE is scarcely involved in affecting the same parameters at the employed concentrations.

**Conclusions:**

The results described in this paper add new important details in the comprehension of the mammalian sperm physiology and suggest new insights for improving reproduction and for optimizing equine breeding.

## Background

Opioid receptors and endogenous opioid peptides form a neuromodulatory system primarily active in the control of nociceptive pathways. Endogenous opioids act as neurotransmitters in both central and peripheral nervous system [1]. They were found in several other districts such as adrenal medulla, pancreatic islets, pituitary, intestinal and bronchial mucosa [2] and in reproductive organs [3-8], thus they seem to be implied in the regulation of nociception, respiration, cardiovascular function, gastrointestinal motility, mood [9] but also in the modulation of the reproductive activity [10-12].

Opioid receptors are also targets of exogenous narcotic opiate alkaloids that constitute a major class of drug abuse [13]. Several reports demonstrate the effects of opiate compounds on sperm cell motility [14-16] and morphology [17] thus suggesting the importance of these receptors in the modulation of reproduction in mammals.

Our current understanding on opioid receptors can be summarized as follows: they belong to the superfamily of seven transmembrane-spanning G-protein coupled receptors [18] and can be divided in three major types named MOR, DOR, KOR (mu, delta, kappa opioid receptors respectively). The opioids/receptor system exhibits impressive diversity in terms of the number of endogenous ligands (more than a dozen) that converge at the three major types of receptors [19]. In general, opioids have been found to inhibit neuronal excitability via two mechanisms: inhibition of calcium and enhancement of potassium conductance [20,21], besides, upon activation, a variety of signal transduction processes are induced with different mechanisms noticeable in diverse cell types such as activation of the mitogen-activated protein (MAP) kinases and the phospholipase C mediated cascade. Moreover, adaptation, which is a consequence of a prolonged exposure to opiates, can cause the internalization of the receptor via a classic endocytic pathway occurring in a ligand specific manner that is independent of ligand ability to stimulate G-protein signalling thus explaining the differences in the efficacy and abuse potential of various opiates [22].

Molecular cloning identified for DOR, as well as for MOR and KOR, an unique gene, even if pharmacological studies indicate the existence of two subtypes for MOR and DOR [23] and at least four subtypes for KOR [24,25]; the existence of multiple mechanisms to achieve distinct pharmacological profiles for receptors originated from a single gene can explain such discrepancy. Even if no mRNA splicing variants have been detected for DOR, several variants at protein level have been identified that are the result of different post translation modifications, such as homo and hetero dimerization with MOR and KOR [26], glycosylations [27,28] likely to be responsible for the appearance of different DOR subtypes [29].

DOR is activated by specific agonists such as cyclo-[D-Pen^2,5^]enkephalin (DPDPE) and [D-Ala2, D-Leu5]-enkephalin (DADLE) and inactivated by selective antagonists like naltrindole and Naltriben [30], as well as by non specific endogenous and synthetic compounds.

DOR has been extensively localized in the central nervous system [31], recently it has been found in human spermatozoa [12] so that its possible involvement in the modulation of sperm motility has been hypothesized. On the other hand it has been demonstrated that sperm cells express MOR [10-12] and KOR [12] and that the human semen contains beta-endorphin, an endogenous opioid peptide [32], Up to now DOR involvement in sperm motility has not been clearly demonstrated: Agirregoitia et al. [12] indeed showed that different doses of DPDPE have no effects on human sperm motility; on the contrary its antagonist, naltrindole, is effective in reducing sperm movements.

Aim of this work is to investigate the relationship between DOR and sperm cell physiology and the possible involvement of DOR in the modulation of viability, motility, capacitation, acrosome reaction (AR) and mitochondrial activity of equine spermatozoa.

## Methods

### Chemicals

Unless otherwise stated, were purchased by Sigma-Aldrich (Milano, Italy), precast Criterion XT gradient 4-12% SDS-polyacrylamide gels were from Bio-Rad (Milano, Italy).

The fluorescent tripeptide: H-Dmt-Tic-Glu-NH-(CH_2_)_5 _-NH-(C = S)-NH-fluorescein, analogue of the delta opioid receptor antagonist Dmt-Tic (Dmt: 2',6'-dimethyl-L-tyrosine; Tic: 1,2,3,4-tetrahydroisoquinoline-3-carboxylic acid) was kindly provided by Prof. G. Balboni (Department of Pharmaceutical Sciences and Biotechnology Centre and Department of Chemistry, University of Ferrara, Italy).

### Animal and semen collection

Gel-free semen was collected in an artificial vagina from six healthy, mature stallions, age 10-12 years, housed at the Equine Reproductive Centre "Pegasus" located at the Faculty of Veterinary Medicine, University of Bari, Italy. The management and the collection of semen samples were performed in accordance with health and welfare regulations in force and approved by the intramural Department Council of the University of Bari.

Semen concentration and motility were measured by the computer assisted sperm analyzer (CASA).

After collection, semen was immediately diluted 1:5 in prewarmed (37°C) Tyrode's medium and washed 3 times at 900 × g. Sperm cells were finally resuspended in Tyrode's medium containing albumin, lactate and pyruvate (TALP) at 5 × 10^7 ^cells/ml concentration.

### Western blot analysis

Cell extracts were prepared by ultrasonic disruption of spermatozoa (8 pulses of 10 sec at 20 kHz, 80 micrometers amplitude) in a cold buffer (0.5% Igepal, 0.1% Tween-20, 8 M Urea, 30 mM N-octyl-β-D-glycopyranoside, 0.5% Triton X-100, 1% Sodium Dodecyl Sulphate, pH 7.2) containing a protease inhibitors mix (1 mg/ml Pepstatin A, 1 mg/ml Leupeptin, 1 mg/ml Aprotinin, 100 mg/ml Phenyl methane sulfonyl fluoride, 100 μg/ml Benzamidine, 8 mg/ml Calpain I and II). After centrifugation (10 min at 5000 × g at 4°C) to remove cell debris, soluble proteins (40 μg/lane) were ran on a precasted 4-12% SDS-PAGE and transferred by the Trans-Blot semi-dry apparatus (Bio-Rad) to an Immobilon-P membrane (Millipore, Milan, Italy). The filter was blocked in 5% not fat dry milk buffer (20 mM Tris-HCl, pH 7.5, 0.15 M NaCl, 1% (v/v) Triton-X100), and proteins corresponding to DOR were identified by the primary antibody (rabbit anti DOR, Chemicon Inc. Temecula, CA, USA) diluted 1:5000 applied for one night. After membrane washing, the secondary antibody (anti rabbit IgG Horseradish Peroxidase conjugated) diluted 1:10000 was applied for 2 h at room temperature and immunoreactive bands detected by the enhanced ECL plus system (Amersham Bioscience, Little Chalfont, UK). A negative control was obtained incubating the filter with a solution in which the primary antibody was absorbed with a molar excess of the immunizing peptide. Moreover to verify an equal protein loading, membranes were stripped by Restore™ Western Blot Stripping Buffer (Thermo Scientific, Rockford, IL, USA) and reprobed with anti β-actin antibody (Chemicon Inc. Temecula, CA, USA) diluted 1:10000.

### Fluorescence detection of DOR

The localization of DOR on equine spermatozoa was investigated by means of a highly selective fluorescent tripeptide analogue of the potent delta opioid receptor antagonist Dmt-Tic [33] and by the same rabbit anti DOR antibody used in the immunoblot detection.

### Fluorescent tripeptide

Equine sperm cells, suspended in TALP buffer, were incubated at 37°C for 30 min in the presence of the fluorescent tripeptide (0.2 nM). In the negative control spermatozoa were first incubated for 15 min with naltrindole (0.2 μM) and then were let react with the fluorescent tripeptide. After incubation sperm cells were fixed in 12.5% glutaraldeide and spotted on a glass slide, mounted with antifade medium (50% glycerol, 0.5% n-propyl-L-gallate, 20 mM Tris pH 8.0) and observed for the fluorescent pattern by confocal laser scanning microscope C1 (Nikon, Japan) using a 488 nm argon ion and a 543 nm helium/neon laser.

### Anti DOR antibody

Five microliter of sperm cells suspension were smeared onto Polysine™ slides (Menzel-Gläser GmbH & Co, Braunschweig, Germany) and let dry to air. Cells were fixed (4% paraformaldeyde/PBS, 5 min) and slides washed (100 mM glycine/PBS) and incubated overnight at 4°C with the same primary antibody (diluted 1:2500 in 1% BSA/PBS) used in the western blot detection. After washing, the secondary antibody, a fluorescein-labeled goat anti rabbit antibody (Sigma-Aldrich, Milan, Italy) diluted 1:200 in Evans Blue/PBS was applied for 2 h at room temperature. As to control, slides incubated with 5% normal rabbit serum in the absence of the primary antibody were prepared. Following the washing step, the slides were mounted with antifade medium as reported above and observed for the fluorescence pattern with Nikon Eclipse E600 microscope (Nikon, Japan) equipped with Nikon Coolpix 990 digital camera.

### Computer assisted sperm analysis

The effects of different concentrations of a DOR agonist DPDPE and antagonist naltrindole, on sperm motility were analyzed by a Sperm Analyzer (CASA-system; HTM-IVOS, Version 12.3, Hamilton-Thorne Biosciences, MA, USA).

Samples containing 50 × 10^6 ^cells/ml in TALP medium were divided in eight aliquots, all of them, except one, were supplemented with the DOR agonist or antagonist at the following concentrations: sample 1, control; sample 2-5 (supplemented with naltrindole 10^-4 ^M, 10^-5 ^M, 10^-6 ^M, 10^-7 ^M respectively); samples 6-8 (supplemented with DPDPE 10^-5 ^M, 10^-6 ^M, 10^-7 ^M respectively) and incubated at 37°C under 5% CO_2_.

From each experimental condition, at time: 0, 30 min, 90 min, 180 min from the drug addition, 2 μl aliquot was recovered and placed in a Leja^® ^4 analysis chamber, 20 micron depth (Leja Products B.V., The Netherlands). The analysis was conducted at 37°C. The following nine motility parameters were measured for each sample: the percentage of motile spermatozoa; the percentage of spermatozoa with a progressive motility; average path velocity (VAP): the velocity of the smoothed cell path (μm/s); straight line velocity (VSL): the average velocity measured in a straight line from the beginning to the end of the track (μm/s); curvilinear velocity (VCL): the average velocity measured over the actual point to point track followed by the cell (μm/s). The software settings recommended by the manufacturer were adjusted as follows to obtain a clear identification of the different spermatozoa: frame rate (Hz) = 60; frames acquired = 45; minimum contrast = 70; minimum cell size (pixels) = 4; medium VAP cut-off (μm/s) = 50.0; threshold straightness (%) = 75.0; low VAP cut-off (μm/s) = 20; low VSL cut-off (μm/s) = 0.0; magnification Optical × 10 = Digital × 1.89; temperature of analysis (°C) = 37; counting Chamber = Leja 4. Ten randomly selected microscopic fields were scanned. After every set of scans, the playback mode was used to analyze the video sequences of the last field, in order to validate whether all spermatozoa were identified and their trajectory reconstructed correctly by CASA system.

### Capacitation and acrosome reaction

Samples analyzed for motility were also assessed for viability, capacitation and AR by the combined Hoechst 33258/chlortetracycline (CTC) assay in order to verify the effects of the different concentrations of DOR agonist/antagonist on the modifications that occur before and during the exocytic event.

A 198 μl aliquot of each sample was examined after 4.5 h of incubation at 37°C in the presence of 5% CO_2_, as previously described [10]. Briefly at each aliquot of sperm cells suspension, a 2 μl of Hoechst 33258 solution was added. The Hoechst 33258 solution was made diluting 1 μl of stock solution (10 mg/100 μl distilled water) in 10 ml of Tyrode's medium. After 2 min of incubation, the dye excess was removed by a centrifugation at 900 × g for 5 min in the presence of 2% (w/v) polyvinylpyrrolidone (PVP) in PBS. The cell pellet was resuspended in Tyrode's medium, an equal volume of CTC solution (0.75 mM CTC, 5mM L-cysteine in chilled buffer containing 20 mM Tris, 130 mM NaCl, pH 7.8) was added together with 8 μl of 12.5% glutaraldehyde. Four microliter of the obtained suspension were placed on a slide with a droplet of antifade and visualized by the Nikon E600 microscope under epifluorescence illumination using 346-460 nm (UV-2A) and 450-630 nm (B-3A) excitation/emission filters for H33258 and CTC respectively. Spermatozoa were classified as: dead when nuclei showed a bright blue fluorescence over the head; live non capacitated when bright green fluorescence was distributed uniformly over the entire sperm head; live capacitated when they showed green fluorescence over the acrosomal region and a dark post acrosome; live acrosome reacted when sperm showed a mottled green fluorescence over the head, green fluorescence only in the post acrosomal region or no fluorescence on the head. At least 200 spermatozoa were scored per slide.

### Mitochondrial staining

At the end of the incubation period (4.5 h), an aliquot of each sperm sample was stained with MitoTracker Orange CMTM Ros (Molecular Probes M-7510, Oregon, USA) to evaluate the effects of the different concentrations of DOR agonist/antagonist on mitochondrial activity. Briefly, in each sample the cell permeant dye was added to reach a final concentration of 0.1 μM and let to react at 37°C under 5% CO_2 _for 30 min. The potentiometric dye is readily sequestered and become fluorescent after oxidation, a process that only takes place under oxidative respiration. Since this process is only relevant in functional mitochondria, this probe is suitable to discriminate sperm with deteriorated mitochondria from aerobically capable sperm [34,35]. After incubation, sperm cells were washed 2 times in PBS, centrifuged in the presence of 2% PVP plus 200 ng of Hoechst 33258 and cell pellet resuspended in PBS. Stained cells were spotted on slides and observed under confocal microscope. Live cells (Hoechst negative) with active mitochondria were visualized with a intensively red fluorescence midpiece.

### Statistical analysis

Statistical analysis of treatments of spermatozoa with different concentrations of DOR agonist/antagonist was performed by one way ANOVA or Chi-square test, and p values of less than 0.05 were considered statistically significant.

## Results

### Western blot analysis

**Figure 1:**
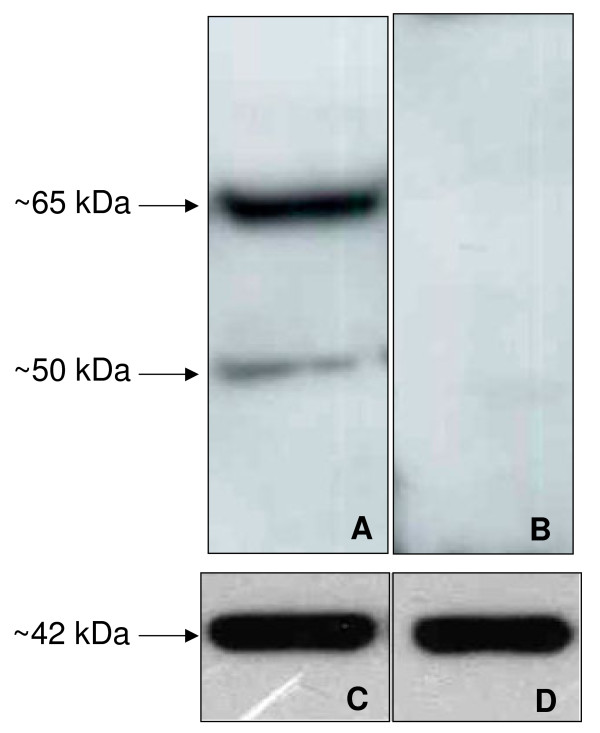
**Western blot for DOR in equine spermatozoa **(A) proteins from ejaculated sperm cells were subjected to gradient 4-12% SDS-polyacrylamide gel and immunoblotted with rabbit anti DOR antiserum. Two bands of 50 and 65 kDa were observed (arrows). (B) negative control: blot containing proteins from ejaculated sperm cells was incubated with a solution of the primary antibody adsorbed with a molar excess of the immunizing peptide. (C and D) same filters re-blotted with rabbit anti β-actin as loading control. Results shown are representative of three separate experiments.

The blot in panel A of figure 1 shows that DOR proteins are detected as ~65 and 50 kDa bands in equine sperm cells; the depletion of the antibody by its immune peptide produces no immunoreactive signals (*Fig *1*, Panel B*). The same filters reprobed with anti beta-actin antibody show that equal amounts of proteins were loaded in the two lanes (*Fig *1*, Panel C, D*).

### DOR expression on equine sperm cells

**Figure 2:**
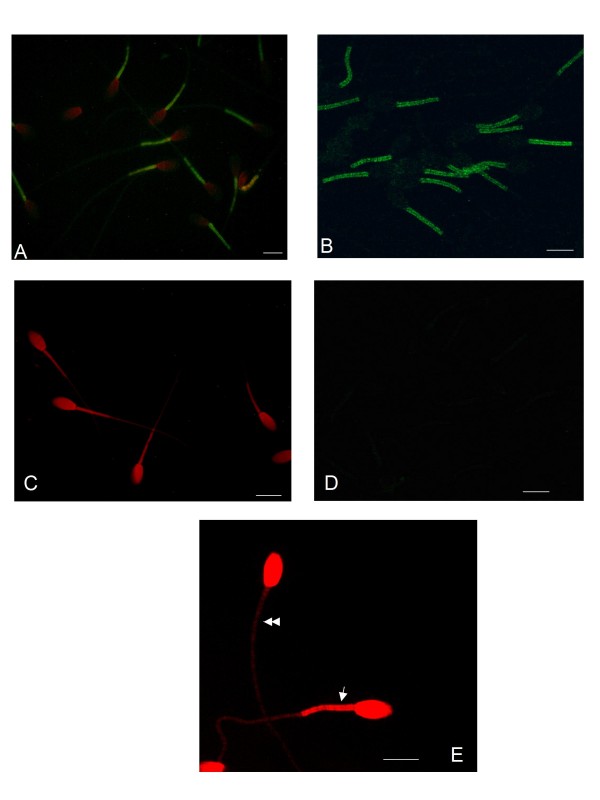
**Localization of DOR and evaluation of mitochondrial activity in equine sperm cells by fluorescence and confocal laser scanning microscopy**. (A) Reactivity staining (green fluorescence) detected by the rabbit anti-DOR antibody is observed exclusively on the mid piece of the sperm tail; sperm cells were counterstained by Evans blue to visualize sperm morphology. Picture was captured by Nikon coolpix 990 digital camera. (B) The highly selective fluorescent tripeptide analogue of the DOR antagonist DMT-TIC detected DOR on the same sperm region; image captured by confocal laser scanning microscope. (C), (D) negative controls for the antibody and the fluorescent tripeptide: in (C), the primary antibody was omitted, only the secondary antibody-FITC conjugated was employed; in (D) spermatozoa were incubated in the presence of 0.2 μmol l^-1 ^naltrindole to block binding sites for the tripeptide. (E) The mid piece of an equine sperm cell containing active mitochondria was intensively labelled by MitoTracker orange (arrow), the cell with inactive mitochondria was only faint coloured in the same region (double arrow). Scale bars, 9.0 μm.

The indirect immunofluorescence approach and the use of the synthetic tripeptide analogue of the DOR antagonist DMT-TIC helped us in the subcellular localization of DOR in equine spermatozoa. With both assays DOR appears exclusively localized in the mid piece of the sperm tail (*Fig*. 2*, Panel A and B*). Negative controls demonstrate the specificity of fluorescent signals (*Fig *2*, Panel C and D*).

### Effects of DOR agonist/antagonist on motility

**Figure 3:**
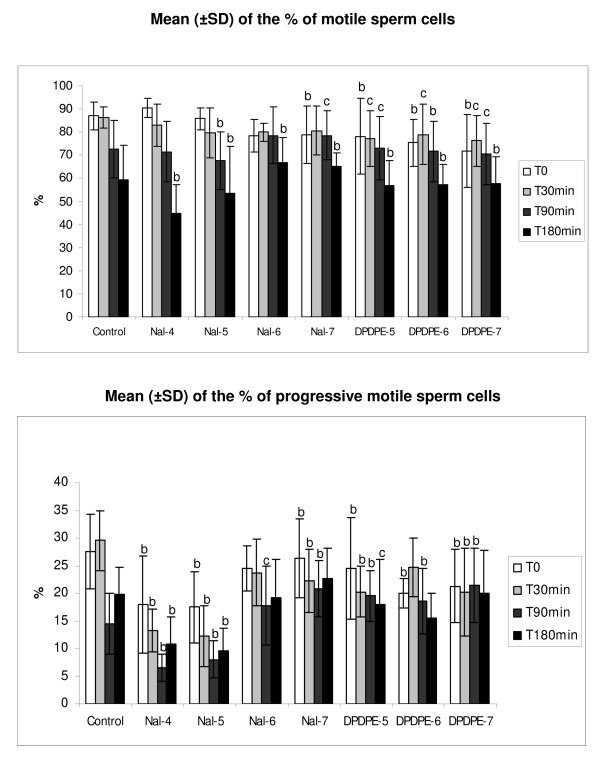
**Dose dependent effects of naltrindole and DPDPE on total and progressive equine sperm cell motility**. Panel A: total equine sperm cell motility affected by different concentrations of DPDPE and naltrindole at T 0, T 30 min, T 90 min and T 180 min. Panel B: progressive equine sperm cell motility affected by different concentrations of DPDPE and naltrindole at T 0, T 30 min, T 90 min and T 180 min. Data are reported as mean ± SD, n = 6. The statistical analysis conducted by Chi square test, after comparison with the control condition at the same time point, was considered significant for p < 0.05. **b **= p < 0.05 and **c **= p < 0.001. Nal-4 = 10^-4 ^M naltrindole; Nal-5 = 10^-5 ^M naltrindole; Nal-6 = 10^-6 ^M naltrindole; Nal-7 = 10^-7 ^M naltrindole; DPDPE-5 = 10^-5 ^M DPDPE; DPDPE-6 = 10^-6 ^M DPDPE; DPDPE-7 = 10^-7 ^M DPDPE.

The agonist DPDPE and 10^-7 ^M naltrindole immediately decrease the percentage of motile sperm cells (p < 0.001; *Fig *3*, Panel A*) after the addition to the culture medium. The agonist keeps the decrease of the motile cells at T30, T90 and T180 min. At T90 min the biphasic effect of the antagonist is noticeable: at 10^-5 ^M it reduces the number of motile cells (p < 0.001; *Fig*. 3*, Panel A*) wherease at 10^-7 ^M these cells are increased (p < 0.05; *Fig*. 3*, Panel A*). At T180 min the biphasic effect is also clear: the reduction of the number of motile sperm cells occurs at 10^-4 ^M and 10^-5 ^M (p < 0,001) whereas at 10^-6 ^M and 10^-7 ^M the increase is visible (p < 0,05; *Fig*. 3*, Panel A*).

### Effects of DOR agonist/antagonist on progressive motility

Immediately after the addition to the culture medium, DPDPE induces a statistically significant reduction in the percentage of cells with progressive motility at all concentrations tested (p < 0.001; *Fig*. 3*, Panel B*). The antagonist naltrindole at 10^-4 ^M, 10^-5 ^M and 10^-7 ^M also decreases the number of progressive spermatozoa compared to the control (p < 0.001; *Fig*. 3*, Panel B*).

After 90 min of incubation the DOR agonist induces an increase of the percentage of progressive motile cells (p < 0,001) compared to the control; after the same time of incubation only 10^-6 ^M and 10^-7 ^M naltrindole increase the progressive motile cells (*Fig*. 3*, Panel B)*. At T180 min no statistically significant difference is recordable for the agonist compared to the control, except at10^-5 ^M; only the highest concentrations of the antagonist (10^-4 ^M and 10^-5 ^M) still reduce the number of progressive cells (p < 0.001; *Fig*. 3*, Panel B*).

### Effects of DOR agonist/antagonist on average path velocity (VAP), curvilinear velocity (VCL), straight line velocity (VSL)

Naltrindole 10^-4 ^M is the only concentration of the antagonist able to modify VAP, VCL, VSL (*Table *1), the agonist at all the concentrations tested doesn't induce a statistically significant variation of the above cited motility parameters (*Table *1).

**Table 1:** Dose dependent effects of naltrindole and DPDPE on VAP, VCL and VSL.

	Control	Nal-4	Nal-5	Nal-6	Nal-7	DPDPE-5	DPDPE-6	DPDPE-7
**VAP at T0min**	128,44 ± 27,21	171,24 ± 27,55^**b**^	151,12 ± 39,52	123,28 ± 18,90	115 ± 30,04	126,54 ± 31,81	120,34 ± 27,60	125,12 ± 32,97
**VAP at T30min**	132,46 ± 10,84	115,56 ± 14,79	124,3 ± 15,68	129,02 ± 15,65	126,98 ± 16,04	129,08 ± 15,18	134,92 ± 12,80	128,16 ± 11,71
**VAP at T90min**	127,84 ± 13,70	106,92 ± 25,54	112,38 ± 24,14	126,36 ± 10,56	125,34 ± 10,34	127,56 ± 9,61	126,26 ± 12,67	128,52 ± 13,48
**VAP at T180min**	99,04 ± 23,63	79,4 ± 19,07	98,28 ± 20,09	113,92 ± 15,14	123 ± 9,89	106,46 ± 20,46	104,56 ± 24,45	123,92 ± 15,36
**VCL at T0min**	224,44 ± 39,23	323,38 ± 55,37^**b**^	277,9 ± 81,77	208,74 ± 21,11	203,9 ± 32,63	218,02 ± 37,39	209,5 ± 30,32	213,32 ± 37,24
**VCL at T30min**	222,9 ± 20,34	231,2 ± 24,26	227,84 ± 27,32	211,44 ± 19,12	215,04 ± 24,88	223,02 ± 14,68	225,68 ± 19,38	214,76 ± 20,07
**VCL at T90min**	228,26 ± 28,49	218,78 ± 46,64	217,68 ± 21,71	215,78 ± 14,78	213,68 ± 16,39	223,14 ± 22,74	215,84 ± 16,97	220,42 ± 24,51
**VCL at T180min**	194,44 ± 37,31	171,7 ± 31,75	199,5 ± 23,51	205,56 ± 25,53	215,14 ± 13,77	200,12 ± 30,24	191,96 ± 31,67	217,86 ± 19,16
**VSL at T0min**	78,06 ± 17,08	81,06 ± 3,27	79,4 ± 10,19	73,58 ± 15,48	68,86 ± 13,72	78,5 ± 21,10	71,74 ± 17,48	76,3 ± 22,68
**VSL at T30min**	83,5 ± 10,45	55,34 ± 9,12^**c**^	68,96 ± 24,11	78,42 ± 18,72	74,14 ± 15,63	75,54 ± 16,42	86,32 ± 15,94	77,5 ± 15,98
**VSL at T90min**	72 ± 11,92	49,76 ± 8,92^**a**^	59,86 ± 28,67	75,12 ± 17,08	73 ± 11,25	81,02 ± 11,78	77,16 ± 17,53	81,66 ± 17,41
**VSL at T180min**	59,94 ± 14,14	37,66 ± 10,00^**b**^	54,02 ± 20,57	66,76 ± 13,58	75 ± 11,88	67,82 ± 19,68	69,32 ± 23,43	76,84 ± 15,94

Table shows the effects of different concentrations of DPDPE and naltrindole at T 0, T 30 min, T 90 min and T 180 min. Data are reported as mean ± SD, n = 6. The statistical analysis conducted by one way-ANOVA test, after comparison with the control condition at the same time point, was considered significant for p < 0.05. **a **= p < 0.01; **b **= p < 0.05; **c **= p < 0.001. Nal-4 = 10^-4 ^M naltrindole; Nal-5 = 10^-5 ^M naltrindole; Nal-6 = 10^-6 ^M naltrindole; Nal-7 = 10^-7 ^M naltrindole; DPDPE-5 = 10^-5 ^M DPDPE; DPDPE-6 = 10^-6 ^M DPDPE; DPDPE-7 = 10^-7 ^M DPDPE.

### Effects of DOR agonist/antagonist on capacitation, AR and viability

Cells recorded as dead have not been assessed for their CTC staining pattern. The addition of naltrindole to the culture medium induces, after 4.5 h of incubation, a statistical significant increase of the percentage of capacitated sperm cells when added at the concentration of 10^-4 ^M (p < 0.05).

**Figure 4:**
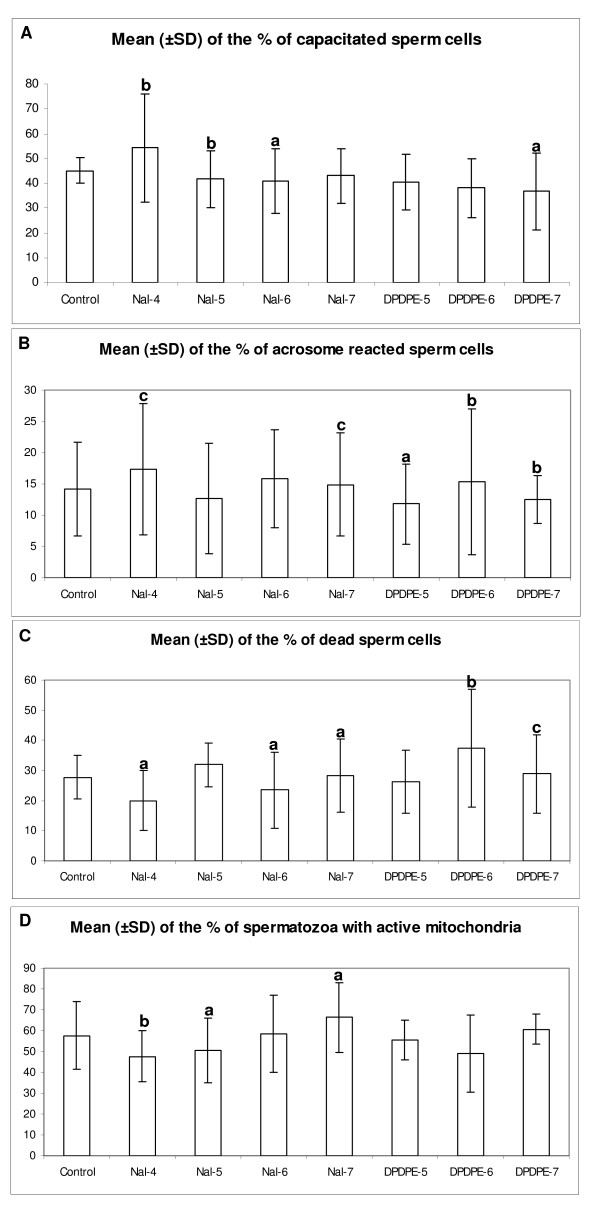
**Dose dependent effects of naltrindole and DPDPE on capacitation, AR, vitality and mitochondrial activity**: Different concentrations of DPDPE and naltrindole affect the number of capacitated (Panel a), AR (Panel B), dead (Panel C) equine sperm cells and those with active mitochondria (Panel D) after 4.5 h of incubation. Data are reported as mean ± SD, n = 6. The statistical analysis conducted by Chi square test, after comparison with the control condition at the same time point, was considered significant for p < 0.01. **a **= p < 0.01; **b **= p < 0.05 and **c **= p < 0.001. Nal-4 = 10^-4 ^M naltrindole; Nal-5 = 10^-5 ^M naltrindole; Nal-6 = 10^-6 ^M naltrindole; Nal-7 = 10^-7 ^M naltrindole; DPDPE-5 = 10^-5 ^M DPDPE; DPDPE-6 = 10^-6 ^M DPDPE; DPDPE-7 = 10^-7 ^M DPDPE.

DPDPE at 10^-7 ^M decreases the percent of capacitated sperm cells (p < 0.01) as well as naltrindole at 10^-5 ^M (p < 0.05) and 10^-6 ^M (p < 0.01) (*Fig *4*, Panel A*).

The DOR antagonist induces an increase of the percentage of AR sperm cells at 10^-4 ^M and 10^-7 ^M (p < 0.001; *Fig *4*, Panel B*); the same increase is observed after the addition of 10^-6 ^M DPDPE, while 10^-5 ^M and 10^-7 ^M significantly decrease the number of AR spermatozoa (p < 0.01 and p < 0.05 respectively; *Fig*. 4*, Panel B*).

Both 10^-6 ^M and 10^-7 ^M DPDPE increase the number of dead sperm cells after 4.5 h of incubation (p < 0.05 and p < 0.001; *Fig*. 4*, Panel C*). The antagonist shows a biphasic effect on sperm viability; at 10^-4 ^M and 10^-6 ^M a reduction in the number of dead sperm cells is observed (p < 0.01, *Fig*. 4*, Panel C*) whereas at 10^-7 ^M an increase in dead sperm cells occurs (p < 0.01).

### Effects of DOR agonist/antagonist on mitochondrial activity

*N*altrindole at 10^-4 ^M (p < 0.05) and 10^-5 ^M (p < 0.01) induces a statistical significant decrease of the percent of sperm cells with active mitochondria (*Fig*. 4*, Panel D*). On the contrary naltrindole at 10^-7 ^M induces an increase (p < 0.01) in the percent of spermatozoa with active mitochondria. No statistically significant effects are induced by DPDPE at the concentrations used in this work. In this study the MitoTracker Orange CMTM Ros has been utilized for the first time on equine sperm cells and it proved to be efficacious for the evaluation of mitochondrial activity. It showed a strong red colour in the mid piece of the tail corresponding to active mitochondria while a faint red colour in the same region, appeared when mitochondria were inactive (*Fig*. 2*, Panel E*).

## Discussion

In this paper DOR has been characterized in equine spermatozoa by both a molecular and a functional approach. The results discussed showed: 1) the presence of DOR in equine sperm cells; 2) its subcellular localization on equine spermatozoa; 3) the relationship between its stimulation by specific agonist/antagonist at different concentrations and some parameters that characterize the physiology of the mammalian spermatozoon such as viability, motility, capacitation, AR and mitochondrial activity.

The delta receptor immunoreactivity following SDS/PAGE appeared as a doublet of approximately 65 and 50 kDa molecular mass bands according with previously reports in other species with slightly differences probably due to the different tissues investigated [36-38]. In human sperm cells Agirregoitia et al., [12] found a unique 50 kDa band while in the brain cortex they found two additional bands of 70 and 35 kDa thus confirming that DOR could be differently expressed in different tissues. The molecular masses evidenced for DOR could arise from post translational modifications of the protein as well as from different transcriptional isoforms of the gene. Further studies are required to clarify this topic.

Immunofluorescence experiments showed that DOR was exclusively localized in the mid piece of the equine sperm tail. This result underlines the involvement of DOR in the physiology of the equine sperm cell; indeed our functional experiments demonstrated that the receptor actively participate in the modulation of motility and in those membrane modifications such as capacitation and AR that make the spermatozoon fertile. The localization of DOR in mid piece of the tail is consistent, in the equine species, with the presence of L-type voltage operated calcium channels [39] and mitochondria in the same cellular district.

Capacitation, AR and motility are calcium dependent events, intracellular calcium increase is due to the entrance of the ion from membrane channels or to its mobilization from intracellular stores such as mitochondria. Our results evidence a clear effect of DOR on the above cellular events indirectly showing a modulative effect on intracellular calcium increase.

The two molecular techniques employed let us hypothesize that different post translational modifications or transcriptional variants of DOR together with its subcellular localization are species specific.

Functional experiments with the use of DOR agonist and antagonist showed that the receptor is actively involved in modulating the kinetic of equine spermatozoa. In this study we employed the same concentration of DPDPE and naltrindole utilized by Agirregoitia et al. [12] in humans to verify whether the different molecular pattern observed in the horse can be responsible for a different pharmacological response of the receptor. The effect exerted by the employed ligands is both immediate and at long term, depending on their dose and on the specific kinetic parameter analyzed. On motility, we observed a reduction of the percent of motile and progressive sperm cells immediately after the addition of the agonist (10^-7 ^M DPDPE). The same effect was observed with higher concentrations of DPDPE on the percent of progressive cells. The antagonist have the same effect causing a dose-dependent decrease in the percent of progressive cells compared with the control. After 180 min of incubation the agonist kept on reducing total sperm motility, whereas the antagonist naltrindole showed an opposite dose dependent effect; indeed motility was decreased at higher concentration (10^-4 ^M and 10^-5 ^M) and increased at the lowest one (10^-7 ^M). The same pattern was shown at 10^-7 ^M naltrindole on progressive cells after 180 min even if the increase induced was not statistically significant probably due to the high standard deviation recorded. Our data support the general concept of the biphasic dose dependent effect displayed by other opioid antagonists [40,41] and confirm previous observations made by our group on MOR [10,42-44]. Our results concerning the progressive motility of the equine sperm cells are not totally in agreement with those reported by Agirregoitia et al. in humans [12], infact, they did not observe any effect induced by DPDPE at any concentration; adding 10^-4^M naltrindole, the unique concentration tested without the association of DPDPE, they found an initial decrease in motility that was restored 3.5 h after the addition.

Other parameters characterized by the CASA system were VAP, VCL, VSL and only 10^-4 ^M naltrindole was able to significantly influence some of these motility descriptors. It is interesting to note that at this concentration it induced a decrease, after 180 min, in VSL and a statistically significant increase in the percent of capacitated and AR cells . These results well correlate with the physiology of the kinetic changes occurring to prepare the spermatozoon to the AR onset. The initial increase of the VSL required for sperm cell progression in the female genital tract, is followed by a decrease of the same parameter due to energy depletion after 180 min. This finding is in agreement with a decrease in the percent of sperm cells with active mitochondria observed 4.5 h after incubation (Fig 4). It is also interesting to note that 10^-4 ^M naltrindole is capable of preserving sperm vitality until 4.5 h of incubation.

Among the concentrations of antagonist utilized, 10^-7 ^M increased the number of AR cells and also that of dead cells, even if the percent of spermatozoa with active mitochondria was increased. This let us suppose that different DOR antagonist concentrations act eliciting different intracellular pathways. The results concerning these specific parameters of the sperm functionality confirm again the biphasic effect showed by high/low concentrations of the DOR antagonist. Naltrindole could be a versatile compound to be used both for in vitro fertilization (IVF) and artificial insemination (AI) it could be employed at high concentration (10^-4 ^M) in IVF protocols because capacitation, AR and viability of spermatozoa are improved, whereas at low concentration (10^-7 ^M) it could be more effective for AI, being able to enhance total and progressive motility and the number of cells with active mitochondria.

The capability of reducing the occurrence of AR can be important in the clinical practice, since the acrosome must remain intact before and during the transit of the sperm to the isthmus until the binding to the zona pellucida has been accomplished. Early acrosome reactions render sperm infertile, and therefore, preserving acrosome integrity before AI is important.

## Conclusions

In conclusion in this study we identified DOR in equine spermatozoa. This result supports the idea that gametes could be a potential target site for endogenous opioids because their specific receptors have been detected both on spermatozoa [10-12] and oocytes [44].

We have also analyzed the effects of DOR activation in the presence of specific agonist/antagonist compounds thus contributing to deepen the knowledge of the equine sperm cell physiology.

We have shown that the DOR antagonist naltrindole can be utilized to modulate specific physiological equine sperm characteristics according to the dose. Low concentrations positively modulate sperm motility; on the contrary high concentrations show the opposite effect. Moreover low concentrations negatively affect capacitation, AR and viability while the opposite effect is exerted by high concentrations. We have also observed a low efficacy of the DOR agonist DPDPE in influencing the same parameters at the used concentrations. In the light of these evidences we can consider naltrindole an eclectic agent capable of different effects on equine sperm cell. These results could have important repercussions on mammalian reproduction and in the economic management of the equine breeding, since the DOR antagonist naltrindole represents a new compound to be used for the improvement of equine semen for AI and IVF.

## Competing interests

The authors declare that they have no competing interests.

## Authors' contributions

MA conceived of the study and coordinated it, performed western blot analysis, cared the statistical elaboration of data and wrote the manuscript. GML helped to draft the manuscript. EM participated in the statistical elaboration of data. ACG carried out immunofluorescence detection, CASA analysis and helped to draft the manuscript. MN carried out semen collection and evaluation. AZ participated in the coordination of the study and helped to draft the manuscript. All authors read and approved the final manuscript.
